# How brazilian parents deal with the development of kids with hearing impairment diagnosis

**DOI:** 10.1192/j.eurpsy.2021.1347

**Published:** 2021-08-13

**Authors:** P. Pacheco, D. Molini-Avejonas

**Affiliations:** Rehabilitation Science, USP, São Paulo, Brazil

**Keywords:** Bioecological Theory of Human Development, hearing impairment, Parenting

## Abstract

**Introduction:**

When parents discover that their child has hearing loss, a new reality presents itself with frustration, a huge amount of work as special care, therapies, exams, etc. Adapting to this new situation is a huge challenge to the development of both parents and children who receive this diagnosis.

**Objectives:**

This study investigated how Brazilian parents of children diagnosed with hearing loss dealt with this situation from diagnosis to the present day.

**Methods:**

In this study it was used the Bioecological Theory of Human Development, which considers the development of both parents and children over time. Two meetings were conducted using a focal group technique, with questions related to the diagnosis and how they faced the situation, prejudice, care of other siblings, etc.

**Results:**

Most parents discovered the diagnosis of hearing loss of their children right after birth. Only one mother said she did not care about the diagnosis of hearing loss while most reported having suffered a lot and glimpsed a life of difficulties. Even knowing the limitations imposed by the condition of the children, no one considered hearing loss as a sickness. Parents reported that the child suffered bullying because of difficulties in speaking and most parents say they worry about their children’s school life.

**Conclusions:**

Parenting kids with hearing impairment is challenging, and involves dealing with prejudice, fear of future, long-term therapies and high costs. Nevertheless parents make great efforts to provide a good environment minimizing the risks of having such condition.

